# Selenium regulation of selenoprotein enzyme activity and transcripts in a pilot study with Founder strains from the Collaborative Cross

**DOI:** 10.1371/journal.pone.0191449

**Published:** 2018-01-16

**Authors:** Roger A. Sunde

**Affiliations:** Department of Nutritional Sciences, University of Wisconsin, Madison, Wisconsin, United States of America; Universidade Nova de Lisboa, PORTUGAL

## Abstract

Rodents and humans have 24–25 selenoproteins, and these proteins contain the 21^st^ amino acid, selenocysteine, incorporated co-translationally into the peptide backbone in a series of reactions dependent on at least 6 unique gene products. In selenium (Se) deficiency, there is differential regulation of selenoprotein expression, whereby levels of some selenoproteins and their transcripts decrease dramatically in Se deficiency, but other selenoprotein transcripts are spared this decrease; the underlying mechanism, however, is not fully understood. To begin explore the genetic basis for this variation in regulation by Se status in a pilot study, we fed Se-deficient or Se-adequate diets (0.005 or 0.2 μg Se/g, respectively) for eight weeks to the eight Founder strains of the Collaborative Cross. We found rather uniform expression of selenoenzyme activity for glutathione peroxidase (Gpx) 3 in plasma, Gpx1 in red blood cells, and Gpx1, Gpx4, and thioredoxin reductase in liver. In Founder mice, Se deficiency decreased each of these activities to a similar extent. Regulation of selenoprotein transcript expression by Se status was also globally retained intact, with dramatic down-regulation of *Gpx1*, *Selenow*, and *Selenoh* transcripts in all 8 strains of Founder mice. These results indicate that differential regulation of selenoprotein expression by Se status is an essential aspect of Se metabolism and selenoprotein function. A few lone differences in Se regulation were observed for individual selenoproteins in this pilot study, but these differences did not single-out one strain or one selenoprotein that consistently had unique Se regulation of selenoprotein expression. These differences should be affirmed in larger studies; use of the Diversity Outbred and Collaborative Cross strains may help to better define the functions of these selenoproteins.

## Introduction

Rodents and humans have 24–25 selenoproteins [1]. These proteins contain the 21^st^ amino acid, selenocysteine (Sec), incorporated co-translationally into the peptide backbone in a series of reactions dependent on at least 6 unique (Se-specific) gene products (reviewed in [2,3]). Glutathione peroxidase (Gpx) was the first identified selenoprotein [4]. That 1973 report also noted that rats fed a selenium-deficient diet had much lower Gpx activity than in Se-adequate rats; subsequent studies showed that liver Gpx1 activity falls dramatically in Se deficiency and can be used to assess selenium (Se) status and requirements [5]. Beginning in the 1990s, a number of researchers reported that there was a “hierarchy of Se regulation” [6], including differential incorporation of ^75^Se into selenoproteins depending on Se status and differential reduction of selenoprotein expression when rodents were made Se deficient [7–12]. That led us to investigate *Gpx1* mRNA level, which we surprisingly found also to be regulated by Se status [13]. Differential regulation by Se status was reported as well for transcripts for selenoprotein P (*Selenop*), 5'-deiodinase (*Dio*), *Gpx4*, *Gpx2*, and other known selenoproteins [8–12]. In our studies, we found that transcripts for selenoprotein W (*Selenow*) and selenoprotein H (*Selenoh*) as well as *Gpx1* were all dramatically down-regulated by Se deficiency in rodents [14–16] and could be used to assess Se status and requirements [17–21], whereas more than half of the selenoprotein transcripts were not significantly affected by Se deficiency in the same tissues.

Lack of the essential Se-containing cofactor readily explains the loss of selenoenzyme activity in Se deficiency, but the mechanism(s) underlying the regulation of selenoprotein transcripts by Se status remain unclear. Synthesis of Sec for incorporation into selenoproteins starts with selenide and with serine [22] esterified to a novel tRNA[Ser]Sec with an anticodon specific for UGA, and proceeds via a phosphoserine intermediate to form Sec-tRNA [2,23]. Selenoprotein transcripts must possess an in-frame UGA codon at the position of Sec incorporation. What distinguishes selenoprotein transcripts from transcripts with a premature or nonsense UGA stop codon is a stemloop SECIS motif (Sec insertion sequence) in the 3'UTR of eukaryotic selenoprotein transcripts [24]. For Sec incorporation, the SECIS element recruits several novel factors that bind the Sec-tRNA, such that the Sec-tRNA out-competes release factors and inserts the Sec into the growing peptide chain. Under Se-deficient conditions, failure to synthesize sufficient Sec-tRNA can make selenoprotein transcripts susceptible to nonsense mediated decay (NMD) [25]. NMD is clearly not the full story, however, as the position of the UGA codon adjacent to the splice junction does not match with the hierarchy of the Se-deficiency regulon [15,26]. Furthermore, transcripts like rodent *Gpx4* mRNA are highly regulated by NMD in cultured cells but not in rat liver [27], demonstrating that other factors are in play. Even relocation of the UGA within the *Gpx1* transcript in cultured cells can affect Se incorporation more than 10-fold, showing that sequence variation has the potential to modify selenoprotein expression [28]. Other proposed mechanisms include a role for differential methylation of the Sec-tRNA anticodon, depending on Se status, which then targets subclasses of transcripts [29], or a role for one of the SECIS binding factors, Secisbp2, in differentially modulating Sec incorporation [30]. The failure so far to better define the underlying mechanism(s) of the Se hierarchy of Se regulation suggests that alternative approaches might be productive.

Considerable effort and progress has been made using genetics and genome-wide association studies (GWAS) to identify genetic differences associated with complex human diseases like obesity and diabetes. A powerful new animal model emerging in tandem with GWAS are studies on the Collaborative Cross [31] and Diversity Outbred strains of mice [32].The Diversity Outbred and the Collaborative Cross collections include 175 lines [33] and at least 69 inbred strains [34], respectively, that were derived from eight Founder mouse strains. The Founder strains and these strains capture approximately 45 million single nucleotide polymorphisms (SNPs) in this population [33]. The use of these animal models is helping to unravel the genetics underlying obesity and diabetes [35] as well as deleterious traits in male reproduction [36] and even polyphenol sensitivity [37]. Published data that included protein expression and transcript expression of selenoproteins in Founder mice consuming standard (Se-adequate) diets reveals relatively uniform, tightly grouped relative expression levels for most selenoproteins [38] and transcripts [39]; much wider variation in expression, however, was reported for protein levels of *Selenow*, and for transcript levels of *Selenow* and *Selenoh* (Supplementary S1 Fig), two of the three most highly down-regulated selenoprotein transcripts in Se-deficient rodent liver. Collectively, this suggested the hypothesis that there could be considerable variation amongst the 8 Founder strains in the hierarchy of Se regulation, driven by global loss or gain of function of factors important in regulation of selenoprotein expression by Se status, or by sequence variation in individual transcripts.

Thus we conducted a pilot study to begin to survey the extent of global variation in regulation by Se status of selenoprotein enzyme activity and selenoprotein transcript expression in the 8 strains of Founder mice. We found that selenoenzyme activity and selenoprotein transcript expression by Se status were globally retained intact, indicating that this regulation is an essential aspect of selenium metabolism and selenoprotein function.

## Methods

This manuscript adopts the new systematic nomenclature of selenoprotein names [40].

### Reagents

Molecular biology reagents were purchased from Promega (Madison, WI, USA), Invitrogen (Carlsbad, CA, USA), or Sigma (St. Louis, MO, USA). All other chemicals were of molecular biology or reagent grade.

### Animals and diets

Young male mice were purchased from Jackson Labs (Bar Harbor, ME, USA) and housed individually in hanging-wire cages. At the start of the study, four strains of mice were 21-days old (B6 (C57BL/6J, Stock 000664); AJ (A/J, Stock 000646); 129S (129S1/SvlmJ, Stock 002448); NOD (NOD/ShiLtJ, Stock 001976)), two strains were 28-days old (NZO, (NZO/HILtJ, Stock 002105); CAST (CAST/EiJ, Stock 000928)), one strain was 35-days old (PWK (PWK/PhJ, Stock 0003715)), and one strain was 49-days old (WSB (WSB_EiJ, Stock 001145)). The basal Se-deficient diet was our rodent torula-yeast diet containing 0.005 μg Se/g diet, supplemented with 100 mg/kg of all-rac-α-tocopherol acetate to ensure prevention of necrosis, and supplemented with 0.4% L-methionine to ensure adequate growth, as described previously [21,41]. Mice were fed the basal diet supplemented with 0 or 0.2 μg Se/g as Na_2_SeO_3_ to provide Se-deficient or Se-adequate diets for 56 days (n = 3/diet for B6, n = 1/diet for other strains), and had free access to feed and water. Body weight was measured twice weekly. The animal protocol was approved by the Research Animal Resources Committee at the University of Wisconsin-Madison (protocol A005368).

### Tissue collection

Mice were killed after 56 days by terminal CO_2_ overexposure followed by exsanguination. Blood was collected in heparinized microfuge tubes. Blood was centrifuged (1500 X g, 15 min, 5°C) to separate plasma from red cells, and the RBC resuspended once, centrifuged, and then reconstituted to original volume using saline phosphate buffer (76 mM NaCl, 50 mM sodium phosphate, pH 7.4). Liver was removed and immediately frozen at -80°C until analysis.

### Enzyme activity analysis

Plasma Gpx3, RBC Gpx1, and liver Gpx1 activity were assayed as described previously [42] with 120 μmol/L H_2_O_2_. Gpx4 activity was assayed with 78 μmol/L phosphatidylcholine hydroperoxide [9]. Thioredoxin reductase (Txnrd) activity was assayed using 5.3 mM 5,5’-dithiobis (2-nitrobenzoic acid) (Sigma D8130), as described previously [43]. The protein concentration was determined by the method of Lowry et al. [44].

### RNA isolation and analysis

Total liver RNA was isolated using TRIzol Reagent (Invitrogen, catalog no. 15596–026) and cDNA libraries were prepared as described previously [15]. Relative mRNA abundance was determined in duplicate by quantitative real-time PCR (qPCR). The mouse gene-specific primer sets for 18 selenoproteins were those used previously and designed to amplify ~150 base segment and to span an intron-exon splice junction when possible [15]. The final 14 μl reactions contained 6 μl cDNA arising from 12 ng total RNA, 6 μl 2X KAPA SYBR FAST qPCR Kit (KAPA Biosystems no. KK4611), and 1 μl each of 10 μM forward and reverse primers. Reactions were followed in a LightCycler 480 (Roche Life Science). Melting curves were generated to confirm the presence of one specific product and standard curves were run for each primer set/tissue combination, and the second derivative max program was used to determine amplification efficiency following the manufacturer’s protocol. mRNA relative abundance was calculated according to Pfaffl [45], accounting for gene-specific efficiencies, normalized to the mean of β-actin (*Actb*) and glyceraldehyde-3-phosphate dehydrogenase (*Gapdh*) expression, and expressed as a percentage of the Se-adequate level. To compare transcript expression of different selenoproteins, relative abundance was normalized for basepair length of the amplified fragment.

### Statistical analysis

Data for B6 mice are presented as mean ± SEM, and treatment differences were analyzed using the unpaired t-test (P<0.05) [46]. Data for Founder mice are presented as box-and-whisker plots (showing middle quartiles, median, and 90 and 10% as error bars) and treatment differences were analyzed using the paired t-test (P<0.05) [46]. Growth rates were compared by ANCOVA (P<0.05) [46].

## Results

**Fig 1:**
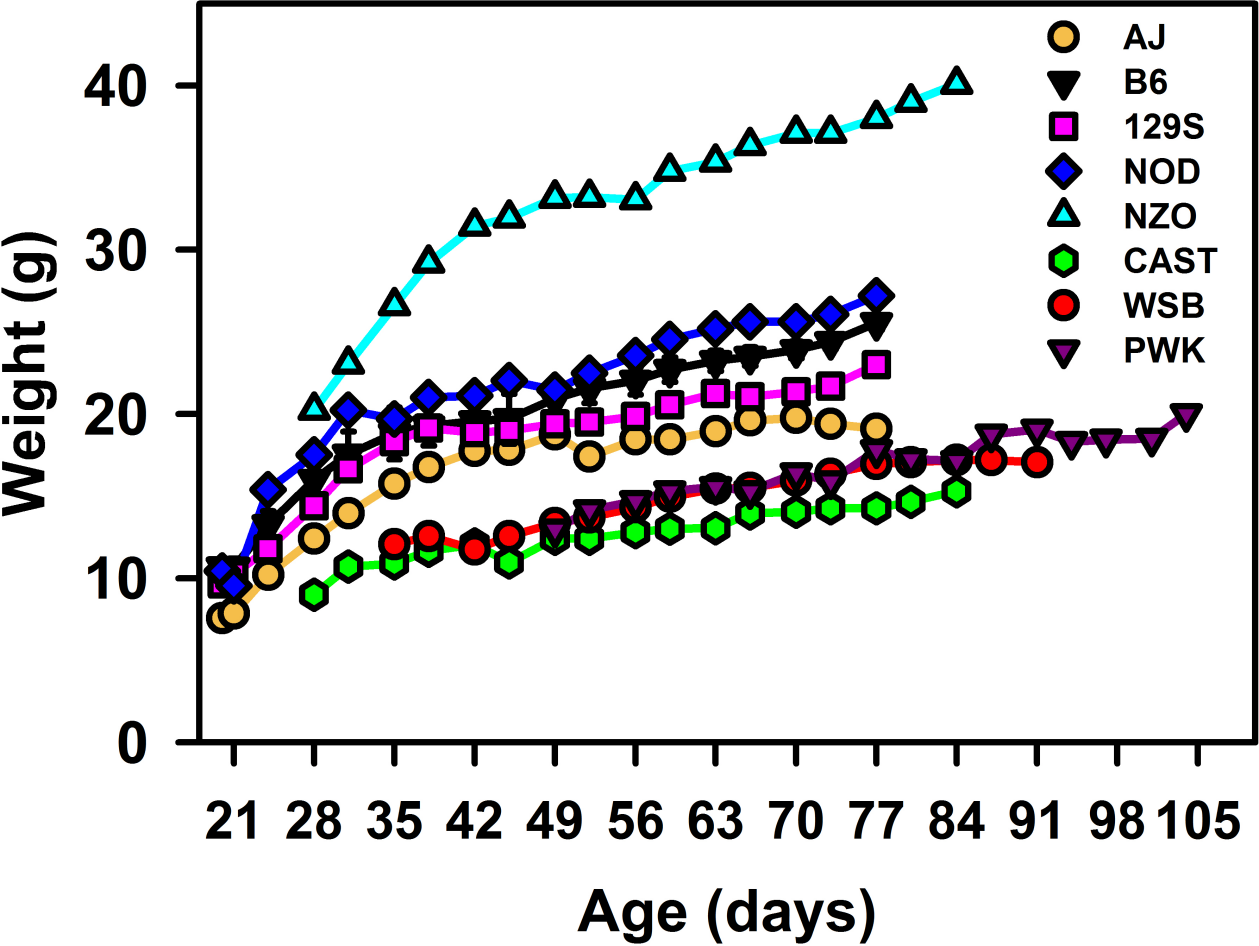
Growth of Founder mice. Eight strains of male mice starting at the indicated age were fed for 56 d, and weighed biweekly. B6 mice (n = 3/treatment), and AJ, 129S, NOD, NZO, CAST, WSB, and PWK (n = 1/treatment) were fed a Se-deficient basal diet (0.005 μg Se/g) or that diet supplemented with 0.2 μg Se/g as selenite. B6 values are the mean weight ± SEM; values for other strains are the mean weight. There was no significant effect (P>0.05) of dietary Se level at any timepoint for B6 mice or for Founder mice.

The study began with four strains of mice at 21 days old, two strains at 28 days old, one strain at 35 days old, and one strain at 49 days old. (Fig 1). There was no effect of dietary Se supplementation on growth of any of the strains (paired t-test P = 0.27). Younger mice showed the characteristic increased growth rate until about day 40. At 49 days of age, CAST, WSB, and PWK mice weighted 59–64% of B6 mice, and weighed 56–70% of B6 mice at day 77. In contrast, NZO mice weighed 158 and 149% of B6 mice at days 49 and 77, respectively. Over the last 4 weeks of Se supplementation, growth rates were linear, and ranged from 0.22 g/d for NZO mice to 0.05 g/d for AJ mice; the growth rate of AJ mice was only significantly different (P<0.05) from the NZO and NOD mice (Supplementary S2 Fig).

**Fig 2:**
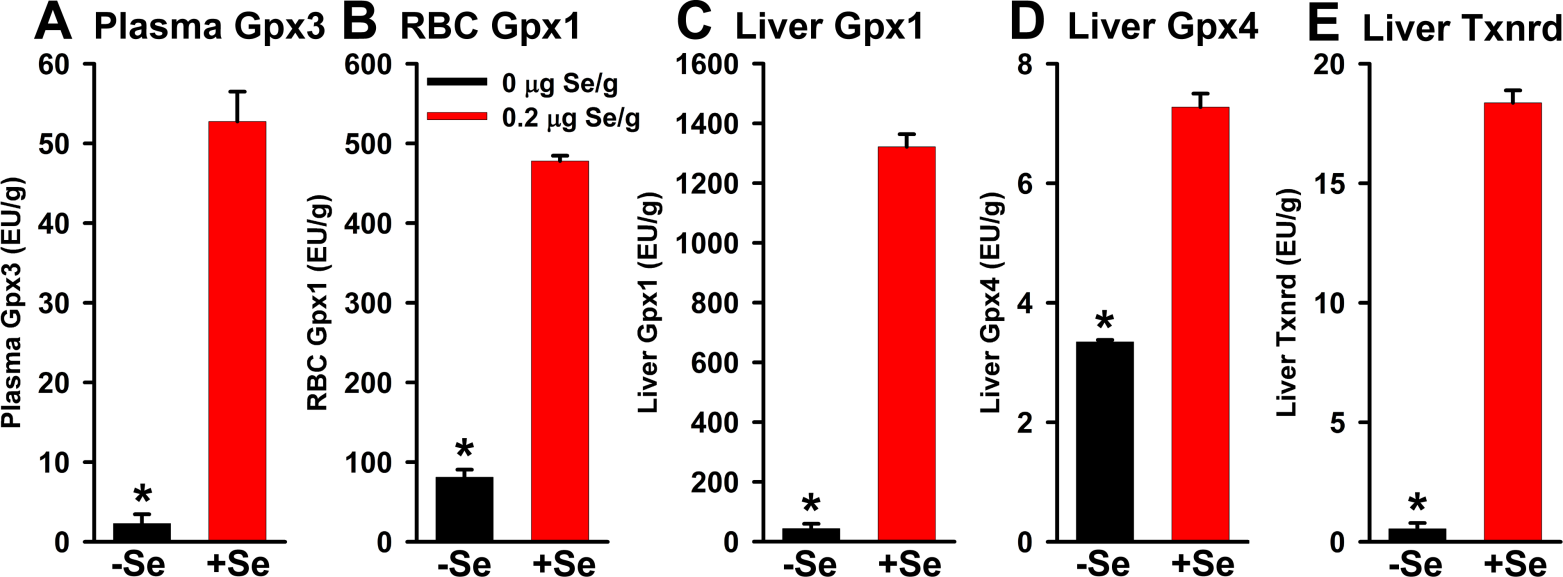
Plasma Gpx3 activity (A), RBC Gpx1 activity (B), liver Gpx1 activity (C), liver Gpx4 activity (D), and liver Txnrd (E) activity in male B6 mice fed 0 (-Se) or 0.2 μg Se/g (+Se) diet for 8 wk. Activities are expressed as enzyme unit/g protein. Values are mean ± SEM (n = 3/treatment); means with asterisks are significantly different from Se-adequate values (P<0.05).

B6 mice were used as the reference strain for this study. When the Se-deficient diet was fed to B6 mice for 8 wk, the levels of plasma Gpx3, liver Gpx1, and liver Txnrd activities fell dramatically and significantly to 4, 3, and 3%, respectively, of levels in mice fed 0.2 μg Se/g diet (Fig 2). Levels of RBC Gpx1 and liver Gpx4 activities were less affected, only decreasing to 17 and 46%, respectively, of levels in Se-adequate mice. These decreases clearly show that these mice were Se-deficient, and the magnitude of the decreases are very similar to previous studies [15,16].

**Fig 3:**
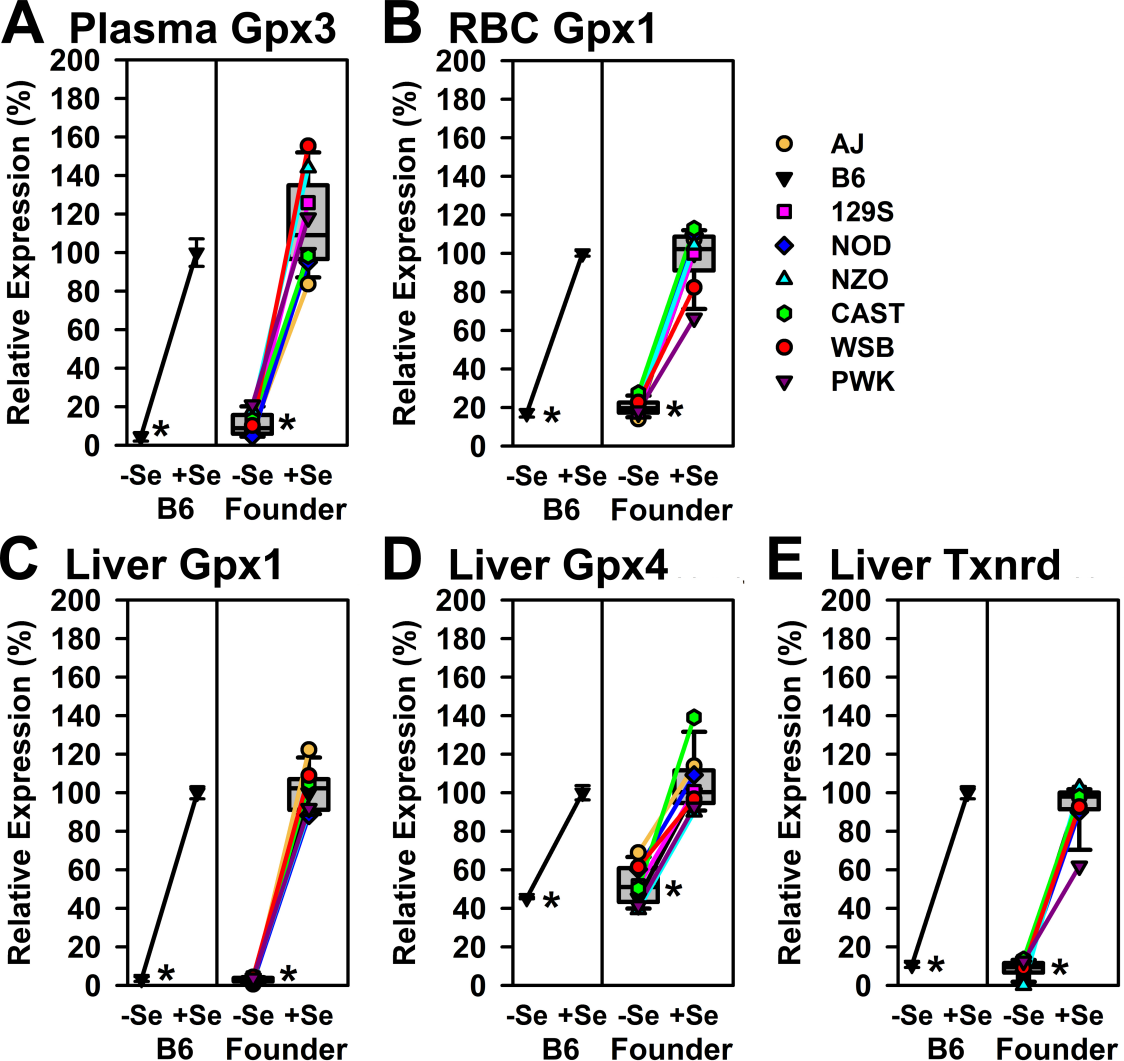
Relative expression of plasma Gpx3 activity (A), RBC Gpx1 activity (B), liver Gpx1 activity (C), liver Gpx4 activity (D), and liver Txnrd (E) activity. Activities are expressed as a percentage of activities in Se-adequate B6 mice. Left portion of each panel shows the relative activity in B6 mice fed 0 μg Se/g (-Se) and 0.2 μg Se/g (+Se) relative to +Se B6 mice; values are mean ± SEM (n = 3/treatment). Right portion of each panel shows the relative activities in -Se and +Se Founder mice as box-and-whisker plots, where the box delineates 25 to 75%, the bar in the box the median, and the error bars 10 and 90%; also shown are individual values linked by lines for each of the 8 strains. Means with asterisks are significantly different from Se-adequate values (P<0.05).

The relative levels of selenoenzyme activity in B6 mice and in Founder mice are shown in Fig 3, plotted relative to levels in Se-adequate B6 mice. Feeding the Se-deficient diet decreased each of these selenoenzyme activities significantly (P<0.05) for all Founder strains as well as for B6 mice. There were no obvious strain outliers for Se-adequate enzyme activity, or for the extent of decrease caused by feeding a Se-deficient diet for 8 weeks to Founder mice. Clearly, Se regulation of expression for these 5 selenoenzyme biomarkers has been retained across all eight of the Founder mouse strains.

**Fig 4:**
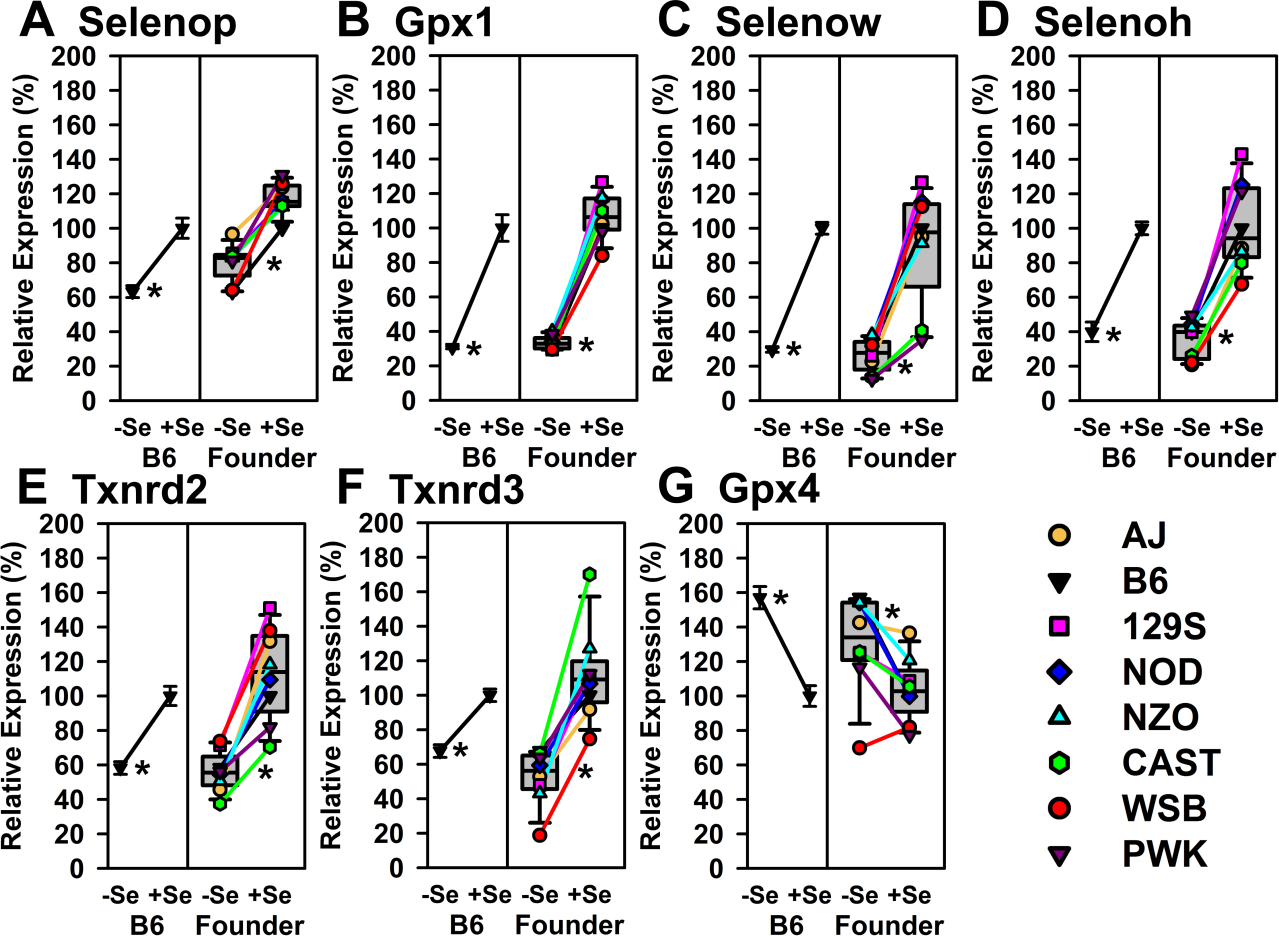
Relative expression of liver selenoprotein transcripts significantly altered by Se deficiency in both B6 mice and Founder mice. Shown are expression levels for *Selenop*, *Gpx1*, *Selenow*, *Selenoh*, *Txnrd2*, *Txnrd3*, and *Gpx4*, expressed relative to the level in +Se B6 mice. Expression is shown as a percentage of levels in Se-adequate B6 mice. Left portion of each panel shows the relative activity in B6 mice fed 0 μg Se/g (-Se) and 0.2 μg Se/g (+Se) relative to +Se B6 mice; values are mean ± SEM (n = 3/treatment). Right portion of each panel shows the relative levels in -Se and +Se Founder mice as box-and-whisker plots, where the box delineates 25 to 75%, the bar in the box the median, and the error bars 10 and 90%; also shown are individual values linked by lines for each of the 8 strains. Means with asterisks are significantly different from Se-adequate values (P<0.05).

Liver selenoprotein transcript expression was determined by qPCR. In Se-adequate liver, the 14 most abundant selenoprotein transcripts were *Selenop*, *Gpx1*, *Gpx4*, *Msrb1*, *Selenok*, *Selenow*, *Selenof*, *Dio1*, *Selenos*, *Selenoo*, *Txnrd2*, *Selenot*, *Txnrd1*, and *Selenoh* (in decreasing order, Supplementary S3 Fig). Other selenoprotein transcripts levels were ~1/2^(9)^ less than (>9 Cp values) the level of *Selenop* expression. In B6 mice fed Se-deficient diet for 8 wk, *Gpx1*, *Selenow*, and *Selenoh* transcripts were the most affected by Se deficiency, falling to 30, 29 add 40%, respectively, of Se-adequate levels (Fig 4). In addition, *Txnrd2*, *Selenop*, and *Txnrd3* transcripts decreased significantly in Se-deficient B6 mice to 58, 63, and 68%, respectively, of Se-adequate levels. Notably in this experiment, liver *Gpx4* transcripts in B6 mice were significantly up-regulated by Se deficiency to 157% of the Se-adequate level (Fig 4G).

The expression of each of the transcripts in this set in Founder mice was significantly regulated by Se status as well (Fig 4). Overall, the patterns of Se regulation were the same as for the B6 mice, with only a couple of lone exceptions; extent of Se regulation of *Selenow* mRNA was reduced for CAST and PWK, and the direction of Se regulation of *Gpx4* was reversed for WSB. *Gpx1*, *Selenoh*, and *Selenow* were the three most down-regulated transcripts, as we reported previously for rats and mice fed Se-deficient diets for 4–5 wk [15,16]. This set of 7 transcripts includes the most abundant transcripts in liver, *Selenop*, *Gpx1* and *Gpx4*.

**Fig 5:**
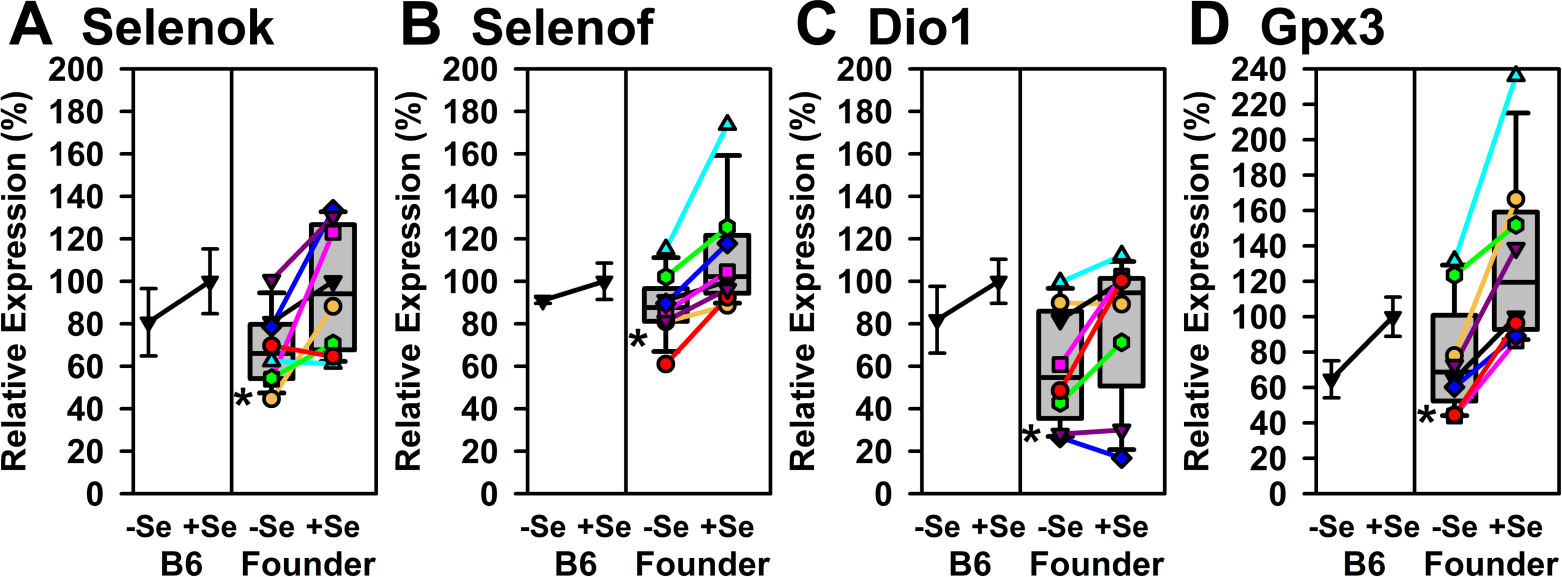
Relative expression of liver selenoprotein transcripts significantly altered by Se deficiency in Founder mice but not in B6 mice. Shown are expression levels for *Selenok*, *Selenof*, *Dio1*, and *Gpx3*, expressed relative to the level in +Se B6 mice. Details as described for Fig 4.

Se deficiency tended to decrease a second set of transcripts in B6 mice, but not significantly. Se deficiency, however, did have a collective significant effect (paired t-test P<0.05) on expression of *Selenok*, *Selenof*, *Dio1* and *Gpx3* in the Founder mice (Fig 5). As with the set of transcripts shown in Fig 4, there were just a few lone exceptions where the extent of regulation (AJ and WSB for *Selenok*, and PWK and 129S for *Dio1*) did not match as well with the other Founder strains.

**Fig 6:**
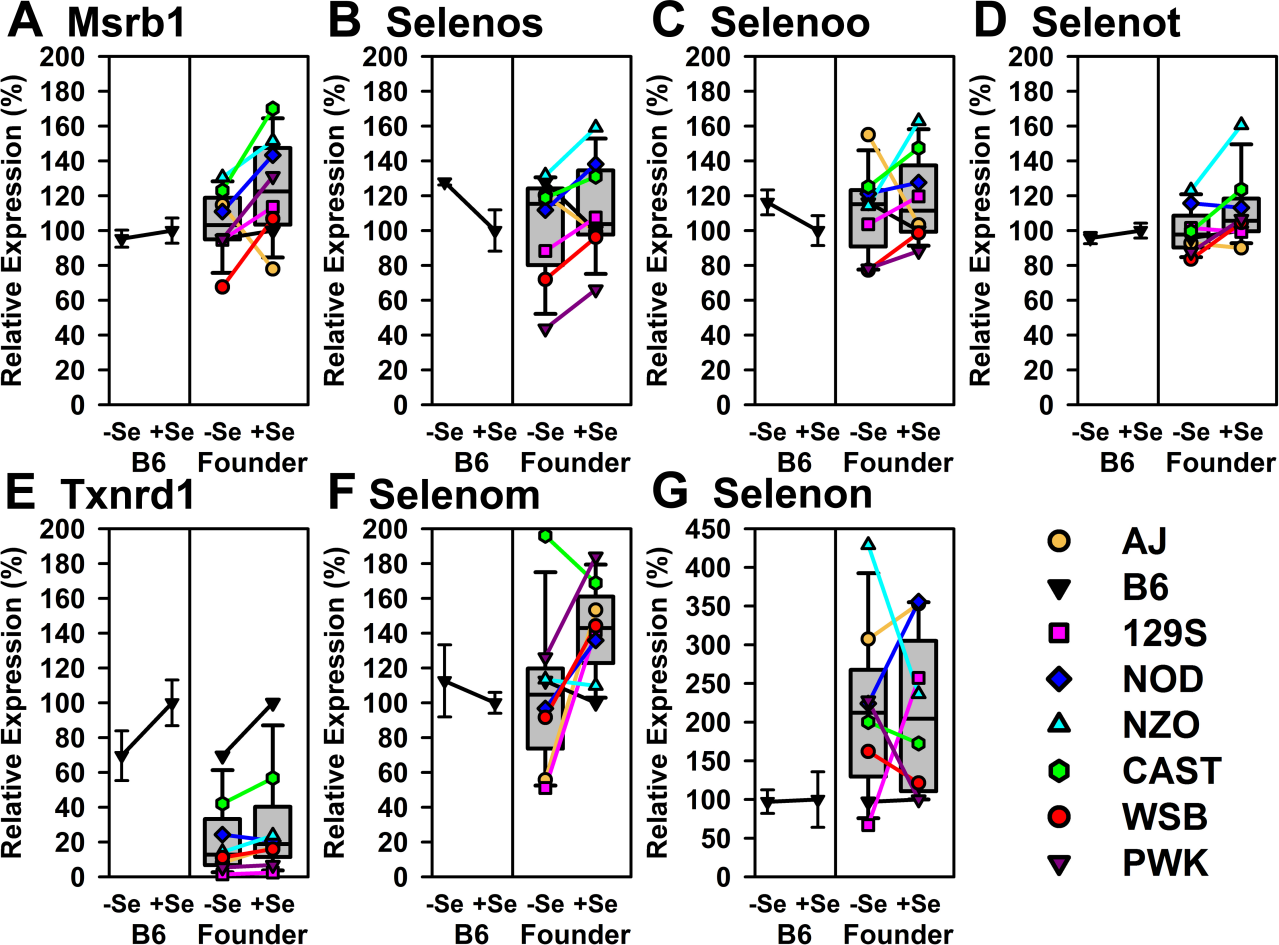
Relative expression of liver selenoprotein transcripts not altered significantly by Se deficiency in B6 or Founder mice. Shown are expression levels for *Msrb1*, *Selenos*, *Selenoo*, *Selenot*, *Txnrd1*, *Selenom*, and *Selenon*, expressed relative to the level in +Se B6 mice. Details as described for Fig 4.

Lastly, the remaining 7 selenoprotein transcripts were not significantly regulated by Se status in either B6 mice or Founder mice in this pilot study (Fig 6). With the exception of *Msrb1*, this set of transcripts consisted of the less-well expressed transcripts, with expression levels <1.5% of the level of *Gpx1* mRNA (Supplementary S3 Fig). We previously reported that *Msrb1*, *Selenos*, *Selenoo*, *Selenot*, *Txnrd1*, *Selenom*, and *Selenon* were not significantly regulated by Se status in mice and rats fed Se deficient diets for 4–5 wk [14–16]. Furthermore, the response to Se deficiency was not consistently up or down amongst the Founder strains for each selenoprotein within this set. The expression level of *Txnrd1* in the Founder strains was less than that observed in B6 mice, and the expression level of *Selenon* and *Selenom* in the Founder strains was higher than that observed in B6 mice, with levels of *Selenon* mRNA as much as 3-4X the level in B6 mice (Fig 6G).

**Fig 7:**
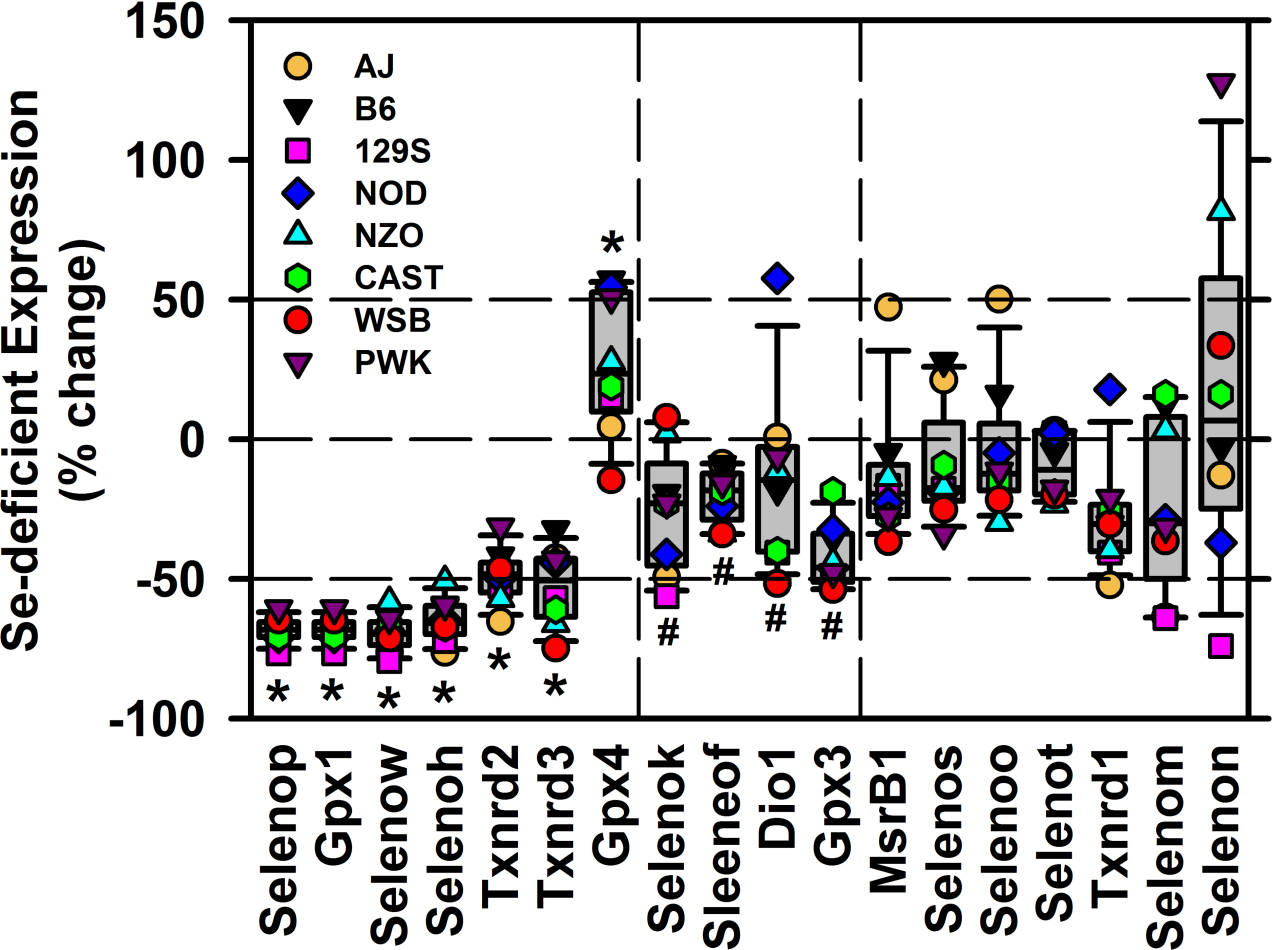
Effect of Se deficiency on selenoprotein transcript expression in Founder mouse liver. Shown are expression levels for 18 selenoprotein transcripts, shown as percentage of expression of that transcript in Se-adequate mice. Box-and-whisker plots indicate the median as the bar in the box, the box delineates 25 to 75%, and the error bars 10 and 90%; also shown are individual values for each of the 8 strains. Horizontal dashed lines separate transcripts regulated significantly both for B6 and Founder mice (*, P<0.05, left), regulated only significantly for Founder mice (#, P<0.05, middle), and not regulated significantly (P>0.05) for B6 or Founder mice (right), determined using t-tests as described for Figs 3–6.

The change in selenoprotein transcript expression due to Se deficiency relative to levels in Se-adequate mice is shown in Fig 7. For the transcripts significantly down-regulated both in B6 mice and in the Founder mice, there were no outlier strains. There was a similar uniformity of Se regulation for 3 (*Selenok*, *Selenof*, *Gpx3*) of the 4 selenoproteins that were significantly regulated in Founder mice but not in B6 mice; *Dio1* expression was nominally up-regulated by Se deficiency in NOD mice. For the set of 7 transcripts not significantly regulated by Se deficiency either in B6 or Founder mice, the median Se regulation, up or down, was <30% (average <24%), and there was no strain that consistently was an outlier up or down for Se regulation of transcript expression. AJ mice were up-regulation outliers for *Msrb1* and *Selenoo*, NOD was an up-regulation outlier for *Txnrd1*, and PWK and 129S were up-regulation and down-regulation outliers, respectively for *Selenon*. Clearly, no strain consistently had the highest or lowest transcript expression relative to the other strains.

## Discussion

Unlike divergent characteristics associated with obesity and diabetes [38,39], Se regulation of selenoprotein enzyme activity and transcript expression was well conserved, and remarkably uniform across these eight strains of Founder mice. Feeding a Se-deficient diet did not significantly alter body weight or growth, when starting with pups from presumably Se-adequate dams and fed these diets for 8 wk. Rodent studies of similar design also typically do not produce growth effects due to Se-deficient diets [14,15]. Note that 21-day old pups from Se-deficient dams fed this Se-deficient diet begin to show reduced growth 7–10 d after the start of the Se-deficient diet [47]. Because we were unable to obtain weanling mice for all strains, mice were fed diets for 8 week, thus doubling the duration of the Se-deficient diet, but also extending the age of the mice to 77 to 105 days old. The results were still rather uniform decreases in selenoenzyme activities, with the level of plasma Gpx3, liver Gpx1, and liver Txnrd activity falling significantly to 3–4% of levels in mice fed 0.2 μg Se/g diet, similar to previous studies [15,16], in both B6 mice and in all Founder strains (Fig 3). Similar, but less dramatic effects of Se deficiency were seen for RBC Gpx1 and liver Gpx4 activities. These results show that Se regulation, at least for these well-studied selenoenzymes, has been uniformly conserved amongst these 8 strains of mice.

We previously reported that *Gpx1*, *Selenow* and *Selenoh* transcript levels in mouse and rat liver are dramatically regulated by Se status, falling to 10–16% of Se-adequate levels in rat liver by qPCR [14], and to 26–32% by qPCR [15] and 7.5–23% by microarray [16] in mouse liver. In the present study with older and thus more slowly growing mice, these transcripts fell to 31–40% of Se-adequate levels in B6 mice. Near identical patterns of regulation of selenoprotein transcripts were observed in all 8 strains of Founder mice for *Gpx1*, *Selenow* and *Selenoh* as well as for *Selenop*, *Txnrd2*, and *Txnrd3*. We did not previously find significant regulation of *Selenop* or *Txnrd3* in mouse liver in studies with 4–5 wk of Se-deficient diet feeding, but did find significant down-regulation of *Txnrd2* [15,16]. Lastly, we sometimes have observed significant up-regulation of *Gpx4* mRNA in muscle in rats [14] or in liver in mice [15] fed Se-deficient diet relative to levels with Se supplementation, but more commonly we do not observed significant regulation in 4–5 wk studies [9,17–20]; in the present study, liver *Gpx4* mRNA was increased significantly by 57% by Se deficiency and by a median of 23% in Se-deficient Founder mice after 8 wk; while WSB and AJ mice did not show this up-regulation of *Gpx4* mRNA, Se regulation of the other 6 transcripts in this set was uniformly down in WSB and AJ mice, similar to the other six Founder strains. Collectively, none of the Founder strains have globally lost the mechanism(s) needed for Se status to regulate selenoprotein transcript expression.

The exceptions and outliers to the general patterns of Se regulation amongst the Founder mice, overall, were few (Fig 7). NOD mice, which are a polygenic model for autoimmune type 1 diabetes, had lower expression of *Dio1* and *Txnrd1* relative to other strains (Figs 5 and 6); Se deficiency, however, resulted in increased expression of *Dio1* and *Txnrd1* relative to the other strains in NOD mice (Fig 7). In contrast, NZO mice, which are a polygenic model of obesity, insulin resistance and type 2 diabetes, were consistently within the 25–75% range of Se regulation by Se status for all but *Selenon* expression. Interestingly, the slower growing AJ mice had elevated expression of *Msrb1* and *Selenoo* relative to other strains when Se deficient, but Se-deficiency appeared to accentuate the decreases in *Selenoh*, *Txnrd2*, *Selenok*, and *Txnrd1* relative to the other strains. Lastly, Se deficiency in WSB mice, a model resistant to diet-induced obesity with low fasting insulin, consistently caused greater decreases in expression of *Txnrd3 Gpx4*, *Selenof*, *Gpx3*, *Msrb1*, and *Selenoo* transcripts than in B6 mice, even though they were 2 wk older at the start of feeding the Se deficient diet, and growing twice as slowly as B6 mice. In addition in WSB mice, there was little apparent effect of Se deficiency on *Gpx4* expression in contrast to the other strains. Similarly, older and slow-growing PWK mice had apparent low levels of *Dio1* and *Txnrd1*, but not *Txnrd2* and *Txnrd3*; PWK as well as CAST strains also did not show as large a change in *Selenow* expression due to Se deficiency as observed in other strains. The suggestion from this pilot study is that these differences are more likely due to overall differences in gene expression amongst these strains which do have differences in expression of genes associated with obesity, diabetes, and other phenotypes [36–39]. Lastly, the response to Se deficiency was not consistently up or down amongst the Founder strains for the selenoproteins shown in Fig 6, especially for *Selenon* and *Selenom*; these transcripts, with the exception of *Msrb*1, were expressed in liver at low levels (S3 Fig), which may account for some of this variation.

Transcript expression, protein expression, and phenotypic expression data are increasingly being reported for these eight Founder strains as well as for the Diversity Outbred and Collaborative Cross mice. Published data on relative liver protein expression in Founder mice shows remarkably tight relative expression for most of the selenoproteins (S1 Fig), although the standard deviations of relative protein expression for *Txnrd3*, *Selenow*, and especially *Dio1* were >25% [38]. Similarly, selenoprotein transcript expression in published data also shows similarly uniform levels for most selenoproteins (S1 Fig), but less so for *Selenom*, *Selenoh*, *Dio1*, and *Gpx3* [39]. Taken together, these data sets indicated that *Selenow* and *Selenoh*–two of the three transcripts dramatically down-regulated by Se deficiency in rodents [14–16]–plus *Txnrd3* and *Dio1*, are transcripts with the most diverse expression differences amongst the Founder strains. The beginning hypothesis for this study was that there would be diversity in Se regulation of selenoprotein transcript expression amongst the Founder strains of mice. The present study, however, clearly shows that the global mechanisms responsible for Se regulation of selenoprotein transcript expression have not been lost within these Founder strains. This indicates that the functions of the 6 unique gene products necessary for Sec synthesis and incorporation have been retained sufficiently to support selenoprotein expression and to support, globally, Se regulation of expression. Furthermore, no one strain stands out with multiple selenoproteins that were differentially regulated in a pattern distinct from the other Founder strains.

In summary, this pilot study demonstrates that the global mechanism(s) that underlie regulation of selenoprotein transcript expression by Se status have been retained intact in all 8 strains of Founder mice. This strongly indicates that this regulation is an essential aspect of selenium metabolism and selenoprotein function. The implication is that the hierarchy of Se regulation is also an important component of the biology of Se. The functions for more than half of the selenoproteins are not known [3], and thus the full impact of the hierarchy of Se regulation remains to be determined. While this survey did not demonstrated loss of global Se regulation of expression, there were apparent differences in expression levels and in response to Se deficiency for individual selenoprotein transcripts. These differences and underlying mechanisms should be affirmed in larger studies; use of the Diversity Outbred and Collaborative Cross strains may help to better define the functions of these selenoproteins.

## Supporting information

S1 Fig Relative selenotranscript and selenoprotein expression in Founder mice. Transcripts expression for 16 liver selenoproteins (A) in the 8 Founder strains is plotted relative to the median level. Shown is the mean of the relative expression of data, as reported Munger et al. [39] (available at http://cgd.jax.org/attie/rnaseq/) for 8 male mice fed standard rodent diet with adequate dietary Se for 26 weeks (NOD mice fed for 20 weeks). Protein expression for 16 liver selenoproteins (B) in the 8 Founder strains is plotted relative to the median level. Shown is the mean of the relative expression of data, as reported by Chick et al. [38], for two male mice fed standard rodent diet with adequate dietary Se for 26 weeks.
(PDF)

S2 Fig Growth of Founder mice during the last 28 days of Se supplementation. Symbols show weights of individual mice, and lines are the least square fit for each of the eight strains. The legend lists the resulting growth rates (g/d); growth rate values not sharing a common letter are significantly different by ANCOVA.
(PDF)

S3 Fig Relative selenoprotein transcript expression in liver of Se-adequate B6 mice. Liver mRNA from B6 mice fed 0.2 μg Se/g diet for 56 days was subjected qPCR for 20 selenoprotein transcripts, *Gapdh*, and *Actb*. Expression is shown relative to *Gpx1*, as corrected for relative oligo length.
(PDF)
