# Supplementary material for: Selenium regulation of selenoprotein enzyme activity and transcripts in a pilot study with Founder strains from the Collaborative Cross
Source: PLoS One. 2018 Jan 16;13(1):e0191449. doi: 10.1371/journal.pone.0191449 (PMC5770059; doi:10.1371/journal.pone.0191449)
Supplement: S1 Fig — Transcripts expression for 16 liver selenoproteins (A) in the 8 Founder strains is plotted relative to the median level. Shown is the mean of the relative expression of data, as reported Munger et al. [39] (available at http://cgd.jax.org/attie/rnaseq/) for 8 male mice fed standard rodent diet with adequate dietary Se for 26 weeks (NOD mice fed for 20 weeks). Protein expression for 16 liver selenoproteins (B) in the 8 Founder strains is plotted relative to the median level. Shown is the mean of the relative expression of data, as reported by Chick et al. [38], for two male mice fed standard rodent diet with adequate dietary Se for 26 weeks. (PDF) [file pone.0191449.s001.pdf]

Supplementary Figure S1

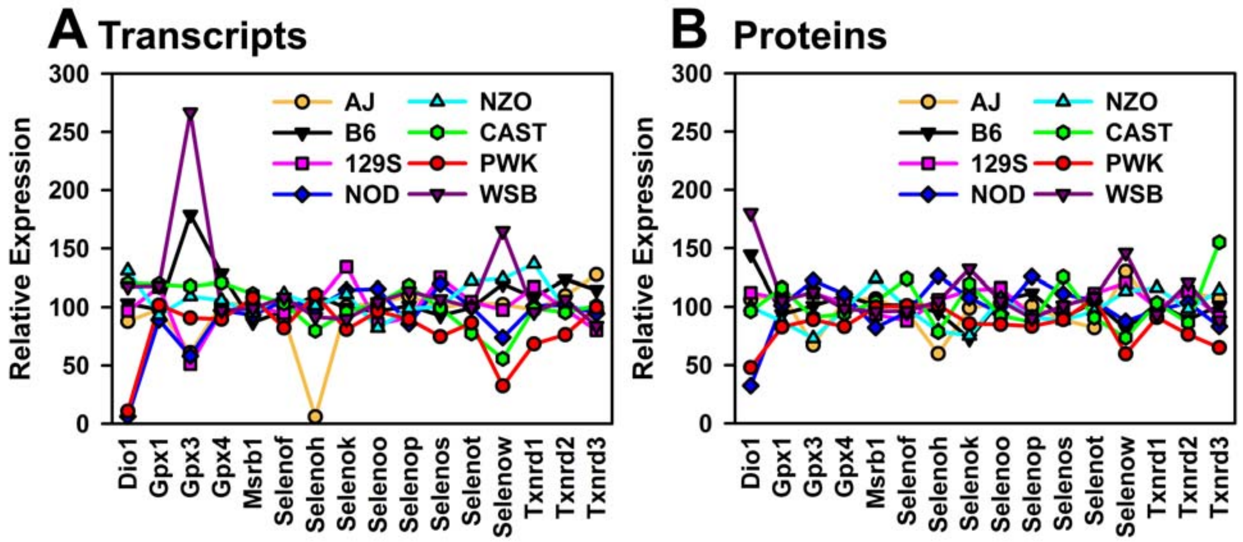

Supplementary Fig S1. Relative selenotranscript and selenoprotein expression in Founder mice. Transcripts expression for 16 liver selenoproteins (A) in the 8 Founder strains is plotted relative to the median level. Shown is the mean of the relative expression of data, as reported Munger et al. [39] (available at http://cgd.jax.org/attie/rnaseq/) for 8 male mice fed standard rodent diet with adequate dietary Se for 26 weeks (NOD mice fed for 20 weeks). Protein expression for 16 liver selenoproteins (B) in the 8 Founder strains is plotted relative to the median level. Shown is the mean of the relative expression of data, as reported by Chick et al. [38], for two male mice fed standard rodent diet with adequate dietary Se for 26 weeks.
