# Supplementary material for: Selenium regulation of selenoprotein enzyme activity and transcripts in a pilot study with Founder strains from the Collaborative Cross
Source: PLoS One. 2018 Jan 16;13(1):e0191449. doi: 10.1371/journal.pone.0191449 (PMC5770059; doi:10.1371/journal.pone.0191449)
Supplement: S2 Fig — Symbols show weights of individual mice, and lines are the least square fit for each of the eight strains. The legend lists the resulting growth rates (g/d); growth rate values not sharing a common letter are significantly different by ANCOVA. (PDF) [file pone.0191449.s002.pdf]

Supplementary Figure S2

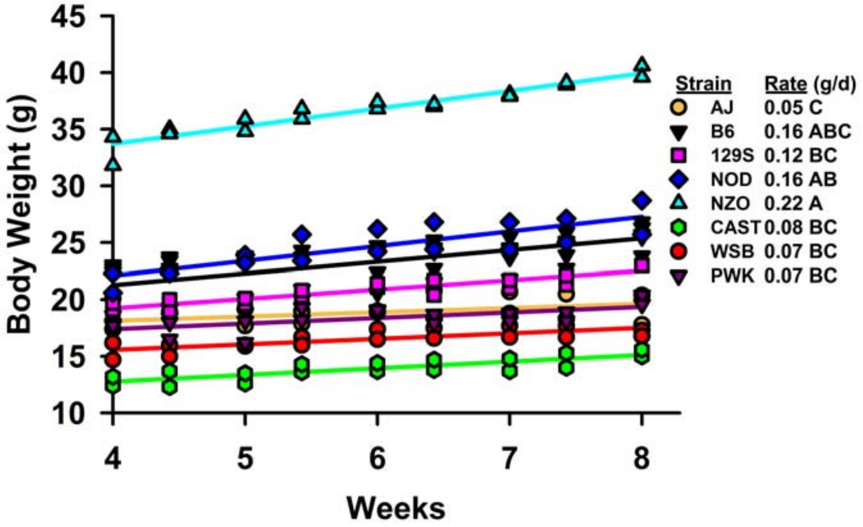

Supplementary Fig S2. Growth of Founder mice during the last 28 days of Se supplementation. Symbols show weights of individual mice, and lines are the least square fit for each of the eight strains. The legend lists the resulting growth rates (g/d); growth rate values not sharing a common letter are significantly different by ANCOVA.
