# Supplementary material for: Selenium regulation of selenoprotein enzyme activity and transcripts in a pilot study with Founder strains from the Collaborative Cross
Source: PLoS One. 2018 Jan 16;13(1):e0191449. doi: 10.1371/journal.pone.0191449 (PMC5770059; doi:10.1371/journal.pone.0191449)
Supplement: S3 Fig — Liver mRNA from B6 mice fed 0.2 μg Se/g diet for 56 days was subjected qPCR for 20 selenoprotein transcripts, Gapdh, and Actb. Expression is shown relative to Gpx1, as corrected for relative oligo length. (PDF) [file pone.0191449.s003.pdf]

Supplementary Figure S3

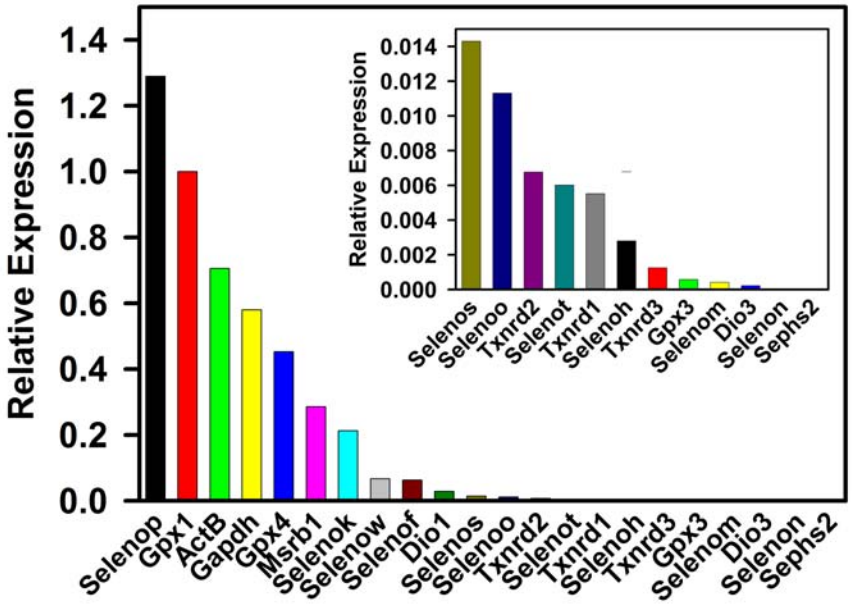

Supplementary Fig S3. Relative selenoprotein transcript expression in liver of Se-adequate B6 mice. Liver mRNA from B6 mice fed 0.2 µg of Se/g diet for 56 days was subjected qPCR for 20 selenoprotein transcripts, *Gapdh*, and *Actb*. Expression is shown relative to *Gpx*1, as corrected for relative oligo length.
